# Predictors of adherence to surveillance cystoscopy for patients with non‐muscle invasive bladder cancer

**DOI:** 10.1002/bco2.70135

**Published:** 2026-02-11

**Authors:** Katherina Y. Chen, Marieke K. Jones, Soutik Ghosal, Grace P. Ignozzi, Stephen H. Culp, Tracey L. Krupski, Jennifer M. Lobo

**Affiliations:** ^1^ Department of Urology University of Virginia Charlottesville Virginia USA; ^2^ Department of Public Health Sciences, School of Medicine University of Virginia Charlottesville Virginia USA

**Keywords:** adherence, cystoscopy, health services, non‐muscle invasive bladder cancer, surveillance

## Abstract

**Objective:**

To identify factors associated with decreased adherence to the national risk‐stratified surveillance cystoscopy schedule for non‐muscle invasive bladder cancer (NMIBC).

**Patients and methods:**

A retrospective, IRB‐approved review was conducted at a single academic institution to identify patients diagnosed with NMIBC who underwent cystoscopy. Patient demographics were collected and driving distance to the urology clinic was calculated. Area Deprivation Index (ADI) and Distressed Communities Index (DCI) were used as proxies for socio‐economic status (SES). Clinical data included year of diagnosis, cancer stage, risk stratification per national guidelines, pathology results and surveillance cystoscopy dates. The primary outcome was 12‐month adherence to surveillance cystoscopy. Stepwise model selection using logistic regression identified factors associated with adherence.

**Results:**

Of 591 patients identified, 351 had a confirmed pathological diagnosis and complete follow‐up data. After excluding 57 patients who experienced recurrence, 112/294 (38.1%) were found to be compliant with the one‐year surveillance schedule. Adherence was inversely associated with travel time to the clinic (OR 0.99, 95% CI 0.99–1.00; *p* = 0.015), while ADI and DCI were not statistically significant in relation to adherence. Other significant predictors included diagnosis before the 2016 guideline update where patients diagnosed pre‐2016 were much more likely to adhere (OR 4.36, 95% CI 2.32–8.55; *p* < 0.001), risk stratification where patients of intermediate‐ and high‐risk were much less likely to adhere than those of low‐risk (intermediate: OR 0.48, CI 0.26–0.88; *p* = 0.018, high risk: OR 0.14, CI 0.04–0.40; *p* < 0.001), and smokers were much more likely to adhere than non‐smokers (OR 1.91, CI 1.08–3.43; *p* = 0.028).

**Conclusions:**

Travel time emerged as a significant barrier to adherence to NMIBC surveillance guidelines, whereas patients' SES did not appear to influence compliance. These findings suggest that logistical obstacles may play a more prominent role than socio‐economic factors. Incorporating telehealth solutions and local partnerships may improve adherence and outcomes for NMIBC patients.

## 1 INTRODUCTION

Non‐muscle invasive bladder cancer (NMIBC) accounts for approximately 75% of newly diagnosed urothelial bladder cancers.^1^ Management is resource intensive for both patients and urologists, largely due to the technical aspect of endoscopic surveillance, frequency of visits and high recurrence rate that may reach up to 78%.^2^, ^3^ This burden is further compounded by a shrinking urologist workforce. Compared to other surgical subspecialties such as neurosurgery, otolaryngology and orthopaedics, the number of urologists per capita has declined, with projections estimating a low of 15.8 urologists per 100 000 individuals aged 65 and older by 2035.^4^ The 2022 American Urological Association (AUA) census identified rural areas as most vulnerable to this shortage, with some counties reporting no practising urologists at all.^4^ The disparity between rural and urban areas in access to care was highlighted at the 2024 American Society of Clinical Oncology meeting, where researchers from the Cleveland Clinic reported significant improvements in bladder cancer mortality in urban settings, but not in rural areas, between 2002 and 2020.^5^ Central to bladder cancer diagnosis and prompt treatment is the cystoscopy, performed by a urologist or urology‐trained advanced practice provider.

Traditionally, patients were told that they required quarterly office cystoscopy, and many continued on this regimen for years. In 2016, the AUA and National Comprehensive Cancer Network (NCCN) introduced updated NMIBC guidelines incorporating a risk‐stratified approach to surveillance that adjusts the frequency of cystoscopy, cytology and imaging based on risk category. This change was in part driven by the economic burden of bladder cancer, which is among the most expensive cancers to manage over a patient's lifetime.^2^ The intense schedule of monitoring, requirement of cystoscopic technology and high recurrence rates lead to a very high lifetime cost of management and treatment. The new 2016 guidelines obviate the need for cytology and imaging for low‐risk patients as well as increase the time between surveillance, all of which lead to overall cost savings. Meanwhile, intermediate‐ and high‐risk patients continue to require cytology at each visit and imaging every one to 2 years. Surveillance cystoscopy schedules now differ by risk group: Low‐risk patients undergo cystoscopy at 3 and 12 months in the first year and then yearly thereafter, intermediate‐risk patients undergo cystoscopy with cytology every 3–6 months for 2 years, while high‐risk patients require cystoscopy every 3 months over the same period.^6^

Various patient‐centric factors can interfere with adherence to this schedule, including logistical, financial and geographic barriers. Non‐compliance can lead to delayed detection of recurrence and missed opportunities to administer timely intravesical therapies, ultimately increasing the risk of disease progression.^7^ Previous studies evaluating compliance with NMIBC guidelines have primarily utilized data from the Surveillance, Epidemiology, and End Results (SEER) database.^8^, ^9^, ^10^ While these studies provided valuable population‐level insights, they may lack the granularity required to assess compliance at the individual level.

The objective of this study was to identify factors, specifically travel time to the clinic and socio‐economic status (SES), associated with 1‐year cystoscopy surveillance adherence among NMIBC patients at a single academic institution with a large catchment area encompassing urban, suburban and rural populations. By understanding these drivers of compliance, interventions may be better designed to address barriers to care and ultimately improve patient compliance.

## 2 PATIENTS AND METHODS

This retrospective study was approved by the Institutional Review Board (IRB# 17573) and conducted at a single academic medical centre. The study cohort included patients diagnosed with NMIBC who subsequently underwent outpatient cystoscopy, identified using Current Procedural Terminology (CPT) code 52000, between 2007 and 2021. Patients were identified through the International Classification of Diseases (ICD) codes: ICD‐10 C67.0 and ICD‐9188.x. To ensure appropriate risk stratification, patients without a verifiable date of pathological tissue diagnosis were excluded. CPT codes 52 231, 52 214, 52 234 and 52 235 (cystoscopy with bladder biopsy) were used to capture office‐based surveillance cystoscopies. We only included patients who had a firm date of confirmed pathological diagnosis (T0) that allowed us to establish both appropriate risk stratification and a baseline surveillance start point. In 2016, the AUA/NCCN guidelines were updated to allow risk‐based adjustments to surveillance cystoscopy intervals, aiming to reduce patient burden and healthcare costs. Prior to this change, surveillance was uniformly recommended every 3 months. Accordingly, we recorded whether a patient initiated care before or after the 2016 guideline revision.

Demographic variables—including age, sex, race, marital status and smoking status—were collected. Due to low representation, non‐White racial groups were combined into a single category. To assess the impact of geographic access to care, patient home addresses were used to calculate estimated travel time to the urology clinic using Google Maps geographic information system (GIS) tools.^11^ Given that many patients resided in rural or underserved areas, socio‐economic status was evaluated using the 2015 Area Deprivation Index (ADI) and the Distressed Communities Index (DCI). ADI incorporates metrics such as income, education, employment, housing quality and other social determinants of health.^12^, ^13^ The ADI was calculated at the census block group level using the patient's residential postal code at the time of diagnosis, with scores ranging from 1 (*least deprived*) to 100 (*most deprived*). DCI is a composite measure of socio‐economic well‐being at the ZIP code level, incorporating economic indicators such as unemployment, poverty rate, business establishment growth, housing vacancy and educational attainment. Scores are grouped into quintiles, with higher quintiles reflecting greater socio‐economic disadvantage.^14^ Clinical variables recorded included cancer stage at diagnosis, AUA/NCCN risk stratification (low, intermediate, high) and pathology results.^6^ Each patient was followed for 1 year from the time of first pathological diagnosis. Patients were excluded if they had T2 or greater disease at initial diagnosis, underwent cystectomy or died within the follow‐up time or if they experienced a recurrence.

### 2.1 Primary outcome

According to AUA/NCCN guidelines, all patients should receive their first surveillance cystoscopy approximately 3 months after initial treatment. For this study, NCCN guidelines were used to define adherence, as they provide a specific 3‐month follow‐up after completing initial evaluation and treatment (vs. the 3‐ to 4‐month window suggested by AUA), which facilitated more consistent outcome measurement. After the initial surveillance cystoscopy, the recommended schedule varies based on risk category. For the 3‐month and 12‐month follow‐up intervals, binary adherence variables were created. Adherence was defined as the completion of a minimum number of cystoscopies within a specified time frame, allowing for a 30‐day buffer before and after each interval to account for scheduling variability, provider availability and facility access.

Low‐risk patients were expected to undergo surveillance cystoscopy at 3 and 12 months. Adherence at 3 months was defined as at least one cystoscopy within 4 months and adherence at 1 year was defined as at least two cystoscopies within 14 months of pathologic diagnosis. Intermediate‐risk patients were expected to undergo surveillance at 3, 6 and 12 months. Adherence at 3 months and 1 year was defined as at least one cystoscopy within 4 months and at least three cystoscopies within 15 months. High‐risk patients were expected to have cystoscopies at 3, 6, 9 and 12 months. Adherence at 3 months and 1 year were defined as at least one cystoscopy within 3 months and at least four cystoscopies within 16 months of diagnosis.^15^

If disease recurrence was detected on surveillance cystoscopy and confirmed by pathology following a transurethral resection of bladder tumour (TURBT) within the study's follow‐up time, the patient was excluded.

### 2.2 Statistical analysis

Descriptive statistics were calculated as frequency and percent for categorical variables and median with interquartile range for continuous variables, stratified by risk group. Associations between covariates of interest and adherence to surveillance schedule were evaluated using Akaike Information Criterion (AIC)‐based stepwise model selection of logistic regression models, with one set of models built for adherence at 3 months and a second set for adherence at 1 year. The full models each included sex, age, marital status, smoking status, cancer stage, AUA guideline year (pre‐ or post‐2016), the DCI quintile, ADI, risk stratification, travel time to the urology clinic and the interaction between risk stratification and travel time. The best model for each follow‐up time was selected based on AIC, which finds the most parsimonious model by seeking the lowest AIC value, representing the best balance between model fit and complexity.

## 3 RESULTS

A total of 591 patients were initially identified from electronic medical records. Of these, 351 had both a confirmed pathological diagnosis and complete medical records 1 year out from their pathologic diagnosis. The majority of patients were male (75%), and the median age was 70 years (interquartile range [IQR]: 64–78). Most patients were White (88%); due to low counts, all other racial groups were combined into a single non‐White category. 70% of patients began receiving care prior to the 2016 AUA/NCCN guideline update. The median driving time to the urology clinic was 49 min (IQR: 27–87) and the median ADI was 39 (IQR: 23–58). Demographic and clinical characteristics, including sex, age, race, ethnicity, marital status, smoking status, timing of care relative to the 2016 guideline update, ADI, DCI, travel time, cancer stage and number of recurrences, are summarized in Table 1, stratified by AUA/NCCN risk category. 29% of patients were classified as low risk, 35% as intermediate risk and 36% as high risk (Table 1).

**TABLE 1:** Characteristics of the patient cohort, stratified by risk category.

Characteristic	Low	Intermediate	High
	*n* = 103	*n* = 121	*n* = 127
Sex			
Male	70 (68%)	96 (79%)	96 (76%)
Female	33 (32%)	25 (21%)	31 (24%)
Age	67 (60, 76)	71 (65, 80)	70 (64, 79)
Race			
White	90 (88%)	107 (89%)	111 (87%)
Non‐White	12 (12%)	13 (11%)	16 (13%)
Unknown	1	1	0
Ethnicity			
Non‐Hispanic	89 (86%)	104 (86%)	110 (87%)
Hispanic	4 (3.9%)	2 (1.7%)	1 (0.8%)
Unspecified	10 (9.7%)	15 (12%)	16 (13%)
Married	67 (65%)	70 (58%)	74 (58%)
Smoking status			
Former/current smoker	73 (71%)	87 (72%)	85 (67%)
Never smoker	30 (29%)	34 (28%)	42 (33%)
AUA guideline year			
Pre‐2016	71 (69%)	79 (65%)	96 (76%)
Post‐2016	32 (31%)	42 (35%)	31 (24%)
Drive to clinic (minutes)	48 (24, 85)	50 (30, 80)	52 (27, 92)
Staging			
Ta	103 (100%)	118 (98%)	36 (28%)
Tis	0 (0%)	0 (0%)	47 (37%)
T1	0 (0%)	3 (2.5%)	44 (35%)
Muscle present	71 (69%)	89 (74%)	102 (80%)
ADI	35 (22, 51)	43 (25, 60)	39 (24, 59)
DCI quintile			
1	37 (36%)	34 (28%)	36 (28%)
2	27 (26%)	26 (21%)	27 (21%)
3	18 (17%)	29 (24%)	34 (27%)
4	8 (7.8%)	15 (12%)	12 (9.4%)
5	13 (13%)	17 (14%)	18 (14%)
Recurrence within 1 year	12 (12%)	23 (19%)	26 (20%)

Abbreviations: ADI, Area Deprivation Index; AUA, American Urological Association; DCI, Distressed Communities Index.

From the sample of 351 patients, 153 (44%) underwent their first surveillance cystoscopy within the recommended 3‐month time frame (including an extra 1‐month grace period). The best model included marital status, smoking status, AUA guideline year and travel time. The strongest predictors of compliance at 3 months were smoking status where smokers were more likely to adhere than non‐smokers (odds ratio [OR]: 1.81; 95% confidence interval [CI]: 1.11–3.00; *p* = 0.019), initiation of care prior to the 2016 guideline change (OR: 1.84; CI: 1.13–3.04; *p* = 0.015), and travel time to the urology clinic (OR: 0.99 per minute; CI: 0.99–1.00; *p* = 0.002) where longer travel times were associated with lower odds of adherence. Marital status was not statistically significant.

**FIGURE 1:**
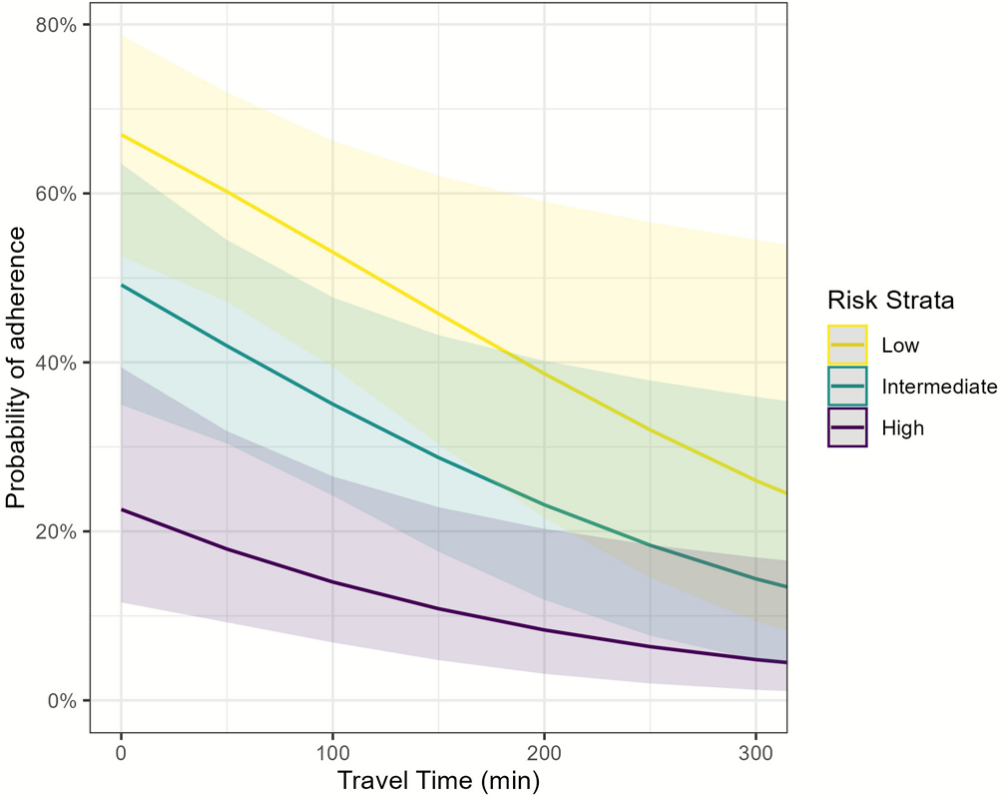
Probability of adherence to surveillance cystoscopy over the first year versus the time needed to drive to the urology clinic, stratified by risk category (low, intermediate, high).

**FIGURE 2:**
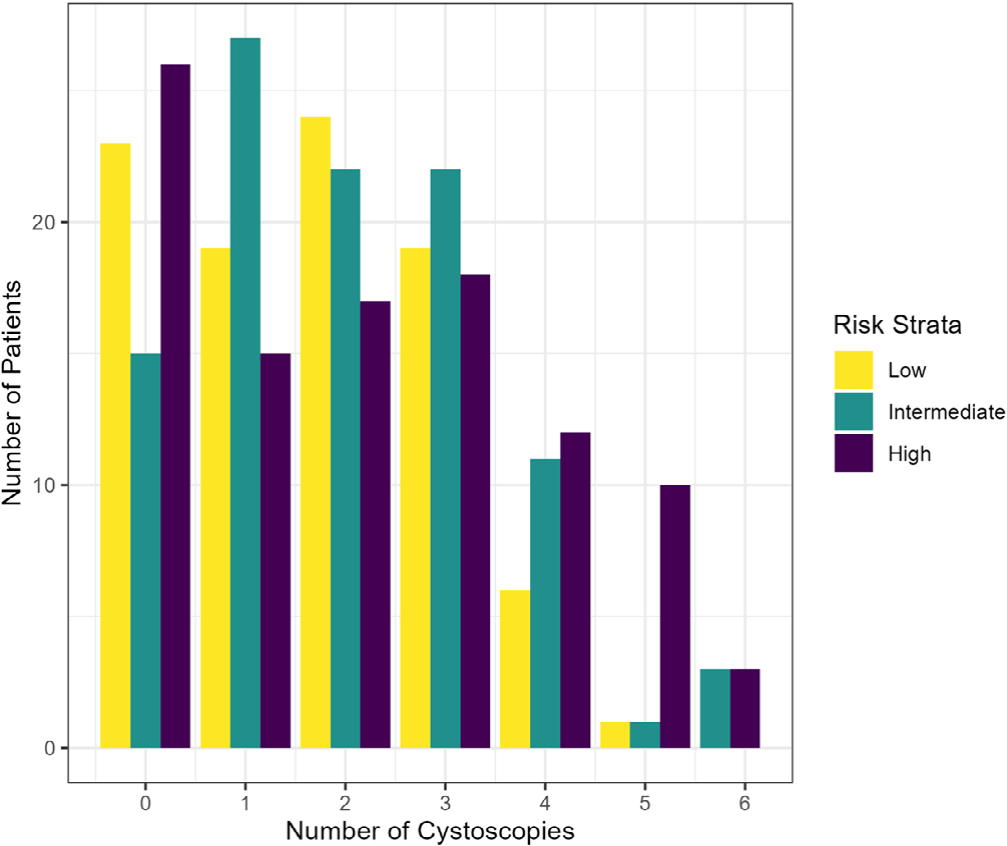
Total number of cystoscopies within the first year (defined as 14, 15 or 16 months depending on low, intermediate or high‐risk category), stratified by risk category.

For 1‐year surveillance, 57 patients were excluded due to recurrence, resulting in an analytical sample of 294 patients. Of these, 112 (38%) were compliant with the AUA/NCCN surveillance cystoscopy schedule. 54% of low‐risk patients, 37% of intermediate‐risk patients, and 25% of high‐risk patients were ultimately compliant. The best model included smoking status, cancer stage, AUA guideline year, risk stratification and travel time. The strongest predictors of 1‐year compliance included current or former smoking status (OR: 1.91; CI: 1.08–3.43; *p* = 0.028), initiation of care before the 2016 guideline update (OR: 4.36; CI: 2.32–8.55; *p* < 0.001) and driving time where longer driving times resulted in lower odds of adherence (OR: 0.99 per minute; CI: 0.99–1.00; p = 0.015) (Figure 1). Additionally, in to low‐risk patients, intermediate‐risk (OR: 0.48; CI: 0.26–0.88; *p* = 0.018) and high‐risk patients (OR: 0.14; CI: 0.04–0.40; *p* < 0.001) were less likely to be compliant (Figure 2). Cancer stage was included in the best model but was not statistically significant. ADI and DCI were not significant predictors of adherence at either surveillance time point.

We did a subsample analysis to determine if providers performing the cystoscopy being oncology trained impacted patient adherence. We randomly selected 50 patients in our cohort and performed chart review to identify the attendings that were performing their cystoscopies. The patients were all largely followed by the same attending until they retired or left our institution and transitioned care to another attending. We found no differences in patient adherence by type of provider.

## 4 DISCUSSION

Surveillance cystoscopies are a cornerstone of care in the management of NMIBC, yet we found disappointing rates of adherence in all groups and an inverse association with cystoscopy based on risk. This is counter to our hypothesis and suggests patient adherence to AUA/NCCN guideline‐recommended intervals can be challenging. In this study of 351 newly diagnosed NMIBC patients between 2007 and 2021, several factors were found to be significantly associated with compliance, including driving time to the urology clinic, the year of diagnosis relative to the AUA/NCCN guideline update, smoking status and risk category. In contrast, demographic variables such as age, sex, race, marital status and SES as measured using ADI and DCI were not significantly associated with patient adherence.

These findings suggest that geographic proximity plays a critical role in compliance. Patients with shorter travel times were more likely to adhere to recommended surveillance intervals, highlighting the importance of physical access to care. This aligns with a prior study that found that a shorter distance to the nearest urological provider was associated with more cystoscopies performed.^8^

Similarly, studies in other disease settings have shown reduced surveillance adherence among patients residing farther away from care facilities: Cirrhotic patients living more than 30 miles from a VA centre were less likely to receive recommended hepatocellular carcinoma imaging and colorectal cancer patients more than 40 miles from a cancer centre were less likely to undergo post‐resection surveillance colonoscopy.^16^, ^17^ A systematic review across various medical conditions further supports the relationship between distance and poorer health outcomes.^18^ These findings reinforce the significance of physical access to care, particularly in conditions like NMIBC that require ongoing, frequent surveillance. The uniformity in these findings presents an opportunity for improvement, suggesting that providing a service within a set geographic radius could improve outcomes. This provision of service could be placement of an urgent care or ambulatory clinic or a collaboration between rural and academic facilities.

The lack of association between the SES proxies (ADI, DCI) and compliance is particularly notable as it challenges long‐standing assumptions that patients of lower socio‐economic status are inherently less likely to adhere to care. This differs from studies in other cancer populations where low socio‐economic status and higher neighbourhood deprivation have been associated with reduced adherence to surveillance follow‐up.^19^, ^20^ This lack of association suggests that when barriers such as travel distance are accounted for, patients across socio‐economic strata may be similarly motivated to adhere to bladder cancer surveillance, perhaps due to the perceived high recurrence risk of NMIBC. This suggests that acting on logistical factors that may be more immediately actionable than on broader immutable socio‐economic determinants could potentially have a larger effect on patient compliance. This also brings an emphasis to our finding that the risk of cancer recurrence was inversely associated with adherence to surveillance. Interestingly, intermediate‐ and high‐risk patients were less likely to adhere to guideline‐recommended surveillance compared to low‐risk patients, despite their higher recurrence risk. This trend did not appear at the 3‐month follow‐up but emerged over the longer term. This also raises a concern that providers are not fully educating patients on the risk stratification and the importance of follow‐up. While counterintuitive, it is not unprecedented. For example, Cha et al. found that patients with low‐risk colonic polyps were more compliant with surveillance guidelines than those with high‐risk polyps.^21^ These findings suggest that higher clinical risk does not necessarily translate into better adherence, possibly due to treatment fatigue, competing health priorities or perceived futility. The work serves a quality control function for our division of urologic oncology to reiterate the rationale of surveillance and how it relates to cancer biology.

The finding that patients that are smokers are apparently more compliant with surveillance cystoscopy is a paradoxical finding since this is a patient group that has historically been found to be non‐compliant with recommended medication use and preventative care for even non‐smoking‐related medical conditions.^22^ However, this finding may be related to the previously discussed patient counselling. Patients that are smokers may get more stringent or harsher counselling since the link between smoking and bladder cancer is so clear such that the enormity of their situation may be better impressed upon these patients than non‐smoking patients, which may have improved the compliance of patients who smoke. Again, this finding reiterates the importance of patient counselling.

The finding that patients diagnosed before 2016 were more likely to comply with post‐2016 surveillance schedules possibly reflects legacy care patterns where more frequent cystoscopy was common across all risk groups. Patients accustomed to frequent cystoscopies prior to the 2016 guideline update may have continued these habits, particularly if their urologists also maintained pre‐2016 follow‐up intervals. These findings underscore the influence of initial counselling and care pathways established at the time of diagnosis. Studies have shown that physician recommendation is a predictor of compliance with colorectal cancer screening, suggesting that adherence behaviours may be influenced as much by provider‐driven follow‐up protocols as by patient demographics.^23^, ^24^ At 1 year, only 38% of patients were adherent to AUA/NCCN surveillance guidelines. The low rate could potentially be explained by patients following up with their local urological provider rather than the tertiary medical centre since the data were obtained from an electronic medical record (EMR) system with CPT and ICD codes, this change in providers would not be apparent. Additionally, miscoding of CPT codes or incomplete documentation could have led to underestimation of compliance. Nonetheless, these findings are consistent with previous studies: Tobert et al. reported only 17% compliance in a similarly rural state, and Chamie et al. found just 1 of 4790 patients was fully compliant with NMIBC follow‐up guidelines.^8^, ^9^

Given the strong association between travel time and adherence, future directions should focus on mitigating geographic barriers. One potential avenue is the development of a tele‐cystoscopy system, where a trained registered nurse performs cystoscopy at a remote site and the video is routed to a tertiary centre where it can be interpreted in real time by a urologist.^25^, ^26^, ^27^, ^28^, ^29^ Expanding satellite clinic coverage with periodic in‐person visits by urologists may also help serve patients in rural or medically underserved regions, though such initiatives have encountered logistical challenges in the past. Notably, a recent demonstration of telesurgery involving a robotic radical prostatectomy performed across international borders suggests the feasibility of remote cystoscopy, which could combine the benefits of the tele‐cystoscopy system with the concept of satellite clinics.^30^ Further research is warranted to assess the feasibility, cost‐effectiveness and patient acceptability of these strategies.

This study has several limitations. As a retrospective analysis, it is subject to the limitations inherent in EMR‐based research. Patients were identified using CPT codes, and compliance was inferred based on documented cystoscopy encounters. We felt the number of cystoscopies captured by our EMR billing underrepresents the perceived number of cystoscopies we perform. However, despite multiple queries and attempts to address this perceived deficit in numbers, we were not able to capture an increased number of cystoscopies. This may have been due to our firm intention to only include patients with a confirmed baseline pathology diagnosis and not those who were established patients on long‐term surveillance. Further, our methodology assumes accurate and complete coding; patients may have been misclassified as non‐compliant due to missing or incorrect procedural codes or if care was received outside the tertiary centre. Additionally, this analysis may not capture more nuanced factors such as provider recommendation strength, patient education level or perceived disease severity, which may also influence patient adherence.

This study suggests that travel time is a key factor influencing adherence to NMIBC surveillance guidelines, while socio‐economic status plays a lesser role than previously assumed. Improving patient compliance with bladder cancer surveillance may require a shift toward addressing modifiable barriers such as geographic access through both telemedicine and outreach clinics or even remote surgery. Innovative models of care delivery may represent a high‐impact strategy to ensure equitable access to guideline‐concordant surveillance for all NMIBC patients.

## AUTHOR CONTRIBUTIONS

SHC, TLK and JML conceptualized the study. KYC, GPI and JML collected patient data and prepared it for analysis. MKJ performed the formal analysis. SG, SHC, TLK and JML provided guidance and oversight on the analysis and interpretation of results. KYC drafted the initial manuscript. TLK and JML acquired funding. All authors critically reviewed, provided edits for, and approved the manuscript.

## CONFLICT OF INTEREST STATEMENT

Dr Krupski and Dr Lobo's work on this project was supported by an American Cancer Society Research Investigator Grant (Grant No. RSG‐16‐161‐01, PI: Dr Krupski). Dr Krupski has subsequently consulted with UROVIU on the development of a prototype remote controlled cystoscope. All other authors report no affiliations with or involvement in any organization or entity with any financial interest or non‐financial interest in the subject matter or materials discussed in this manuscript.
